# Culturally contextualized suicide prevention for international students: new opportunities for research and practice

**DOI:** 10.3389/fpsyg.2024.1418185

**Published:** 2024-08-06

**Authors:** Samuel McKay, Jocelyn I. Meza

**Affiliations:** ^1^Orygen, Parkville, VIC, Australia; ^2^Centre for Youth Mental Health, The University of Melbourne, Parkville, VIC, Australia; ^3^Department of Psychiatry and Biobehavioral Sciences, University of California, Los Angeles, Los Angeles, CA, United States

**Keywords:** culturally responsive, foreign students, college students, university students, study abroad

## Abstract

The rising incidence of suicide-related thoughts and behaviors among international students presents a significant public health challenge and growing concern among college campuses. Current intervention strategies often rely on Western-centric and colonized approaches developed and tested with primarily Western, Educated, Industrialized, Rich and Democratic (WEIRD) samples. Exclusion and historical underrepresentation of ethnoracially minoritized groups in suicide prevention treatment trials create gaps in advancing our science because they often miss the cultural contextualization crucial for effective prevention and intervention in diverse groups from different countries of origin. To address the limitations of these Western-centric strategies, we explored existing prevention recommendations and approaches through the lens of an expanded version of the newly developed Protective Factors Framework, tailored for non-Western cultural perspectives. We propose significant opportunities for enhancing current practices and point to promising future directions. The primary areas for development include: (1) bolstering community empowerment and ownership, (2) refining mechanisms of change to encompass multicultural viewpoints, and (3) focusing on effective implementation and thorough evaluation for ongoing refinement. This methodology not only shows promise for enhancing international student suicide prevention but also offers insights for broader application in suicide prevention among other culturally diverse populations.

## 1 Introduction

Suicidal thoughts and behaviors among international college students in key destination countries such as Australia and the US represents a significant public health challenge (Veresova et al., 2024). Existing evidence suggests current prevention and intervention strategies are, at best, only partially effective (Jamieson, 2019, 2021; McKay et al., 2023). In such countries, prevention strategies primarily employ Western mental health paradigms focusing on individual-level interventions. Indeed, a comprehensive review of suicide prevention strategies for international students revealed that current approaches primarily seek to enhance mental health literacy, facilitate access to local services, and heavily rely on symptom reduction (McKay et al., 2023). The foundational evidence for such approaches largely stems from studies involving participants from Western, Educated, Industrialized, Rich, and Democratic (WEIRD) societies (Maria Guzmán et al., 2024), which are not representative of the majority of international students (UNESCO, 2024). Research with diverse cultural groups has demonstrated that such strategies overlook cultural and other contextual considerations crucial to effective prevention and intervention (Wexler and Gone, 2012; Alvarez et al., 2022; Look et al., 2023).

In this article, we propose that integrating culturally contextualized prevention models, which blend evidence-based Western mental health best practices with the unique cultural perspectives of the international student population, can enrich existing efforts and unveil innovative opportunities for prevention and intervention. We begin the article by overviewing existing evidence on international college student suicidality before turning to the literature on culturally responsive suicide prevention. Building on this research, we examine the existing recommendations for prevention and intervention among international students. Our analysis leverages recent theoretical developments from the Protective Factors Framework (Allen et al., 2022), while also drawing upon established models including the public health model of suicide prevention (World Health Organization, 2021a, 2022; Pirkis et al., 2023). This approach allows us to identify critical areas along the suicide care continuum where culturally responsive approaches have significant potential to enhance the effectiveness of suicide prevention efforts for international students.

### 1.1 Suicide risk and help-seeking among international students

Suicide is a leading cause of death for young people aged 15–29 (World Health Organization, 2021b), and rates are particularly elevated in students enrolled in post-secondary education such as university or college (Mortier et al., 2018). International college students represent a large proportion of post-secondary students in many countries (In 2021: Australia = 22%, Canada = 17%, UK = 20%, U.S. = 5% OECD, 2023), and suicidal thoughts and behaviors are common among this group (Veresova et al., 2024). Evidence predominantly from the U.S. indicates varying rates of suicidality among international students across different contexts. In general student populations, international students show similar past year suicidal ideation (5.6–9.8% vs. 5.2–13.3% for domestic students) and self-harm (4.3–17.2% vs. 3.2–22.9% for domestic students 3.2–22.9%; Goodwill and Zhou, 2020; Yeung et al., 2021; Xiong and Pillay, 2022; Zhou et al., 2022). However, concerning trends emerge when examining more severe outcomes. In general student populations, international students report higher rates of past year suicide attempts (1.21–2.2%) in comparison to domestic students [0.1–1.6% (Goodwill and Zhou, 2020; Yeung et al., 2021; Xiong and Pillay, 2022)] and this disparity is even more pronounced in clinical settings. For instance, among students seeking psychiatric services, international students show a higher lifetime history of multiple attempts for those seeking psychiatric services (international = 14.3%, domestic = 6.5%; Hong et al., 2022). This is particularly troubling, as suicide attempts are a significant risk factor for future suicide attempts and deaths (Ribeiro et al., 2016). Despite these elevated rates of suicide attempts, there is a scarcity of research that uncovers the risk and protective factors associated with suicide-related thoughts and behaviors among international college students (Veresova et al., 2024).

Risk and protective factors play crucial roles in understanding and predicting suicide-related outcomes (Fonseca-Pedrero et al., 2022). Risk factors are characteristics or conditions that increase the likelihood of suicidal thoughts or behaviors, while protective factors are elements that reduce this risk and promote resilience (Fonseca-Pedrero et al., 2022). These factors interact dynamically, with protective factors potentially mitigating the impact of risk factors (Allen et al., 2022).

Research on the risk and protective factors for suicidality among international students can be grouped into three main areas, many of which are associated with acculturation challenges arising from adapting to a new culture, education system, and country (Orygen, 2020; Veresova et al., 2024). The first set of risk factors include experiences of social isolation, with loneliness, unmet personal needs, and low social support and campus belongingness all related to increased suicidal ideation and self-harming behaviors (Servaty-Seib et al., 2016; Taliaferro et al., 2020; Nguyen et al., 2021; Low et al., 2023). The second category includes intrapersonal factors (i.e., individual level factors) such as depression, anxiety, maladaptive perfectionism, hopelessness, and low problem-focused coping, which have been shown to increase risk for suicidal ideation and self-harm (Yang and Clum, 1994; Wang et al., 2013; Jun et al., 2022; Low et al., 2023). Lastly, contextual factors, including perceived discrimination, academic and life stress, and unmet family expectations of academic performance, are each related to suicidal ideation, with perceived public stigma, particularly amongst Asian international students, predicting suicide attempts (Goodwill and Zhou, 2020; Taliaferro et al., 2020; Pérez-Rojas et al., 2021; Low et al., 2023). Many of these factors can also be protective when reversed. For instance, high levels of problem-focused coping and social and university connectedness serve as buffers against suicidality when experiencing stressful life events (Low et al., 2023). Other protective factors include greater cultural sanctions against suicide and family cohesion in the form of high warmth and low conflict (Taliaferro et al., 2020). While research has identified these factors, numerous other potential risk and protective factors for international students have yet to be tested in relation to suicidal thoughts and behaviors despite being identified as predictors of poor mental health and implicated in international student suicide deaths.

Key predictors of poor mental health among international students include language difficulties, acculturation issues, cross-cultural loss, and financial stress (Zhang and Goodson, 2011; Minutillo et al., 2020). Although published research on international student suicide deaths is scarce, several coronial investigations into such cases in Australia have revealed that a variety of stressors, including those outlined above, often are linked to death by suicide (Jamieson, 2019, 2021; McGregor, 2023). These investigations also highlight that students often did not seek or engage with university or mental health support services before the suicides occurred.

Research into the barriers facing international students in seeking help and utilizing services indicates that cultural stigma, limited mental health literacy, and structural obstacles like unavailable, unaffordable, or culturally misaligned services can significantly hinder help-seeking and service utilization (Lu et al., 2014; Clough et al., 2019; Jamieson, 2021). Cultural stigma regarding mental health concerns relates to both perceptions of weakness or “craziness” associated with mental health issues along with potential consequences for individual or family reputations (Ma et al., 2021). Similarly, international students often report concerns regarding potential consequences such as losing their visa or academic issues if they report mental health issues (Orygen, 2020). Poor symptom recognition can also contribute to the problem, with many international students unaware that their experiences and symptoms are representative of mental health problems (Lu et al., 2014). Lastly, even when international students overcome these issues, they are more likely to drop out of treatment than domestic students, and it has been suggested that this is because available services are often not aligned with cultural expectations (Bartholomew et al., 2022).

### 1.2 Culturally responsive approaches to suicide prevention

There is increasing interest in culturally responsive suicide prevention approaches for migrant and ethnoracially minoritized communities (Wexler and Gone, 2012; Na et al., 2016; Bowden et al., 2020; Allen et al., 2022; Meza and Bath, 2022). Culturally responsive suicide prevention can be broadly defined as prevention activities centered on the unique cultural, linguistic, and belief systems of different groups to enhance the effectiveness of suicide prevention efforts (Wexler and Gone, 2012). Recommended culturally responsive approaches by Meza and Bath (2022), which follow the Public Health Model of Suicide (World Health Organization, 2021a), include the following key components: (1) gathering large surveillance data that accurately reflects prevalence rates among diverse communities, in this case, international college students (Xiong and Pillay, 2022); (2) contextualizing risk and protective factors to include systems-level and culturally pertinent elements, such as experiences of discrimination or culturally significant values [e.g., community centered approaches (Allen et al., 2022)]; (3) incorporating culturally relevant risk and protective factors into the development of new treatment strategies [e.g., family discrepancy in academic performance expectations (Jun et al., 2022)]; and (4) engaging ethnoracially diverse minority communities in developing and testing the feasibility, acceptability, and ultimately the effectiveness of prevention and intervention methods (Allen et al., 2022). A growing body of evidence shows that culturally sensitive and responsive approaches are a promising direction for preventing suicide among migrant and non-western cultural groups (Villarreal-Otálora et al., 2019; Sjoblom et al., 2022).

A recent test of the Cultural Theory and Model of Suicide demonstrated that cultural risk and protective factors (e.g., acculturative stress, cultural sanctions, etc.) significantly influence suicide attempts beyond classically tested factors like hopelessness, depression, and reason for living in minoritized groups (Chu et al., 2019). Similarly, reviews on the experiences of depression and the varying language and idioms used to express distress across different cultures reveal distinct patterns in the understanding and expression of such disorders in non-Western contexts (Kaiser et al., 2015; Haroz et al., 2017). Such views are often associated with stigma toward both suicide and formal help-seeking (Bowden et al., 2020), leading to a preference for self-reliance and informal support systems like family networks, which could also provide valuable alternative avenues of support (Na et al., 2016). However, these supports may also be problematic when such networks are not readily available to international students living in another country, or if people within the network react negatively to the person’s help seeking.

Culturally responsive approaches address these unique aspects by avoiding the pitfalls of Western-centric assumptions that can make prevention efforts ineffective or harmful (Wexler and Gone, 2012). By prioritizing cultural congruence (i.e., matching interventions to the target population’s cultural needs), these approaches seek to create prevention activities aligned with relevant cultural norms and perspectives (Wexler and Gone, 2012). While many of these approaches already acknowledge the importance of protective factors, there is a growing emphasis on more explicitly integrating cultural strengths (i.e., racial/ethnic identity pride) into prevention efforts (Allen et al., 2022; Bath and Meza, 2023). This evolution represents a shift from historically deficit-focused approaches to those that more intentionally leverage cultural strengths, marking an important advancement in the research, prevention, and intervention strategies for minoritized groups and international students (Yakunina et al., 2013; Anandavalli, 2021; Allen et al., 2022).

Building on this evolving approach to cultural strengths, much of the research on culturally responsive strengths-based approaches stems from suicide prevention efforts with American Indian and Alaskan Native (AI/AN) populations. A notable recent advancement in this work relevant to international students is the culturally responsive Protective Factors Framework (Allen et al., 2022). This framework refines our understanding of emphasizing the importance of distinguishing between protective factors and protective mechanisms to understand how they interact to confer resilience against suicide risk factors (Allen et al., 2022). In this framework, protective factors are characteristics in an individual or the environment that reduce the risk of suicide. In contrast, *protective mechanisms* are the interrelated sequelae that mitigate risk and promote health outcomes. For instance, social support is a protective factor that may operate through multiple mechanisms. For instance, one mechanism could be reducing emotional distress, while another could be fostering a sense of belonging to promote resilience against suicidality. Separating these protective factors (e.g., social support) and mechanisms (e.g., sense of belonging) facilitates more nuanced perspectives on how prevention activities can confer resilience by mitigating risk factors.

Importantly, the framework also emphasizes the need to consider how each person’s sense of continuity in the past, present, and future is intrinsically intertwined with the analogous features of their broader community and culture. This approach places culture as central to prevention and implementation, encouraging community engagement and ownership of such efforts. Each of these elements makes the Protective Factors Framework ideally suited to supporting the development of a more culturally responsive suicide prevention approach for international students.

## 2 A more comprehensive approach to suicide prevention with international students

The existing literature with a variety of ethnoracially minoritized, migrant, and culturally and linguistically diverse groups suggests that a contextualized, strengths-based approach to suicide prevention with international students will likely support better outcomes (Na et al., 2016; Healey et al., 2017; Kirmayer and Jarvis, 2019; Allen et al., 2022). Specifically, by drawing on the strengths of international students while also acknowledging their cultural needs, two primary areas of opportunity for developing and enhancing suicide prevention emerge. Firstly, a culturally contextualized approach can help identify the unique risk and protective factors, mechanisms of change, and treatment gaps that impact international college student suicide. Secondly, this approach can also support the development of novel prevention initiatives that draw upon students’ cultural strengths, capital, and networks. The possibilities of each of these are outlined below.

### 2.1 Integrating culturally responsive models to improve current prevention approaches

As described earlier, while no evidence-based suicide prevention programs for international students are currently available, a wide range of recommendations exist that can guide research and practice (McKay et al., 2023). Several of these recommendations seek to better meet the cultural needs of international students through adapted risk screening, service provision, and communication tools. However, numerous opportunities exist to improve current host nation services, interventions, and research programs through a culturally contextualized approach, particularly when building upon the Protective Factors Framework.

**Figure 1:**
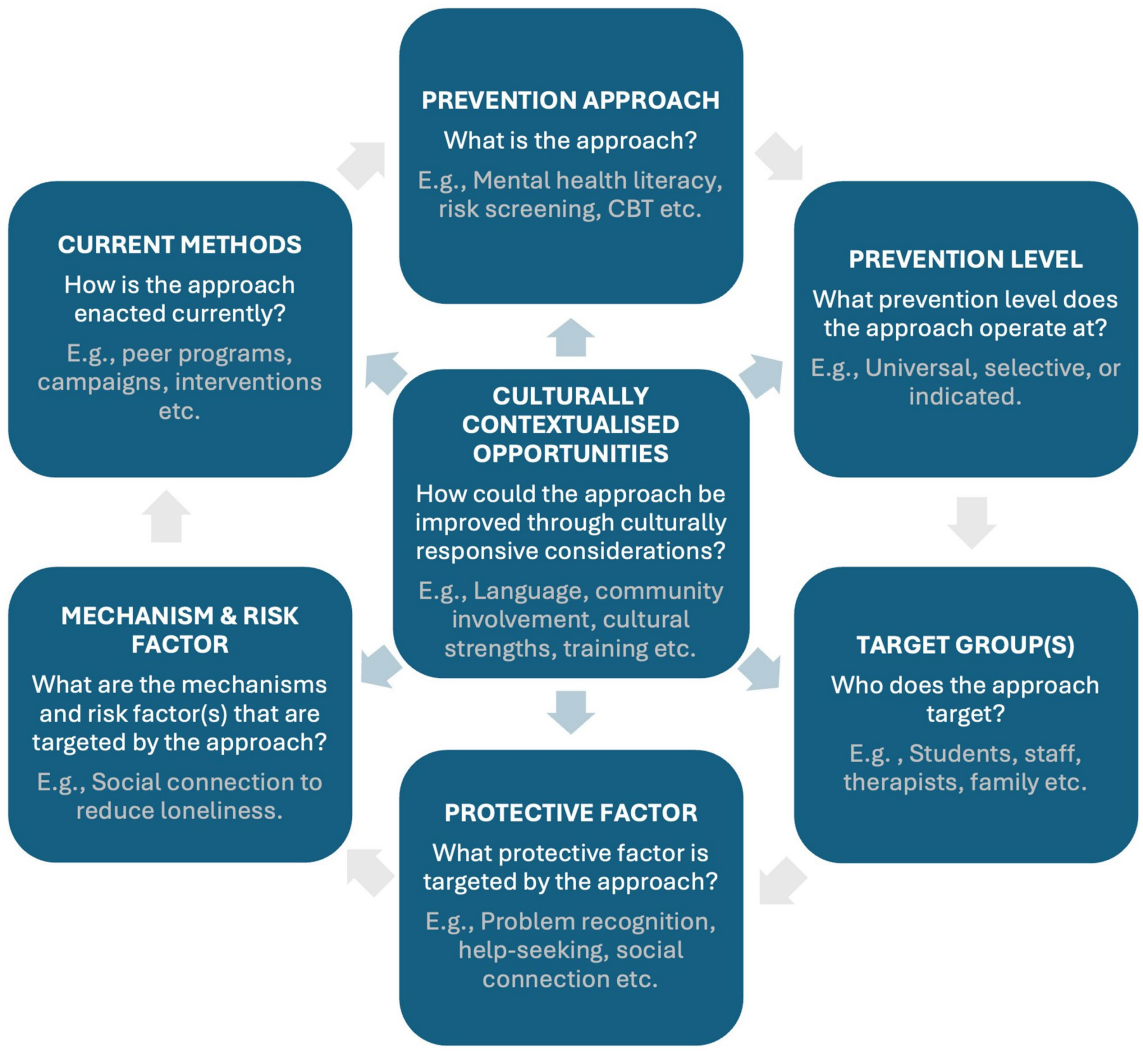
Extended framework for culturally contextualized suicide prevention.

In our proposed integrated model, we have also expanded upon this framework to include three other important considerations relevant to suicide prevention in education settings with international students. Firstly, we have incorporated a well-established public health prevention framework that categorizes prevention efforts according to the level of risk within the targeted population (World Health Organization, 2021a, 2022). The three levels include universal interventions targeting the whole community regardless of risk, selective interventions for those potentially at risk of suicide, and indicated interventions for those demonstrating suicide-related thoughts or behaviors. Secondly, we have added the target groups for each approach to delineate whether it targets students, staff, or other relevant groups. Incorporating prevention levels and target groups helps to better identify gaps in existing approaches. Lastly, we have added culturally contextualized opportunities to show how current prevention efforts could be improved through taking a culturally contextualized approach to prevention. Figure 1 presents a comprehensive overview of the extended framework, illustrating the iterative process of the key components, while also providing guiding questions and relevant examples for each element.

Based on findings from a recent scoping review that reported recommended suicide prevention approaches for international students (McKay et al., 2023), we have summarized current recommendations and practices within this extended framework in Table 1. In the table, each general recommendation is outlined along with the level of prevention, protective factor, mechanism and associated risk factor, target group of approach, current practice methods, and potential avenues for culturally contextualized enhancements.

**Table 1:** Current suicide prevention recommendations for international students and opportunities for development through culturally contextualized adaptations.

Recommended approach	Level of prevention	Target groups	Protective factor	Mechanism and associated risk factor	Current methods	Culturally contextualized opportunities
Mental health literacy campaigns targeting common international student stressors	Universal	All international students	Problem recognition	Increasing students’ awareness of signs of mental health problems	Mental health literacy programs such as contact campaigns, community-led initiatives, and digital interventions, including those that aid with symptom recognition, ideally in multiple languages	Increased involvement of international students with lived experiences in program development and delivery. Mentorship programs or work opportunities with associated training to help train and support those with lived experience to take ownership of the programs. Include cultural strengths and resilience factors in campaign materials, highlighting non-western coping mechanisms for stress and adversity
Mental health literacy campaigns targeting service awareness	Universal	All international students	Help-seeking and formal support	Increasing service engagement by addressing poor awareness of available local services or supports	Information campaigns before arrival, during orientation, and through general promotional materials and campaigns throughout the college experience	Enhanced recognition of diverse mental health perspectives, expanded peer-led initiatives, utilization of multilingual and culturally tailored resources, and engagement through culturally relevant communication channels both online and offline
Mental health literacy campaigns targeting stigma	Universal	Students, families, and community members with shared cultural backgrounds	Help-seeking and Informal or formal support	Increasing willingness to seek help through reducing stigma toward suicide both at individual and community levels	Stigma reduction programs such as contact campaigns, community-led initiatives, and the development of culturally specific programs that use non-stigmatizing language (e.g., excessive thinking)	Drawing upon existing evidence from other migrant and culturally and linguistically diverse groups to identify effective approaches, language, and implementation strategies. Identify opportunities for community ownership and input in program development and refinement
Community risk screening by gatekeepers	Universal	International students, college staff, resident advisors, student leaders, and those who work with international students	Help-seeking and formal support	Increasing service engagement through gatekeepers identifying suicide risk and referring those at risk who may not otherwise seek help to appropriate services	Gatekeeper training programs	Culturally adapted programs or the development of new programs designed to address international student experiences, cultural perspectives, and needs
Mentorship or “Buddy” programs	Universal	All international students but can be culturally specific where appropriate	Social connection	Reduced sense of loneliness by creating social connection	Current students are linked with arriving international students through formal buddy programs organized by education provider	Offering culturally adapted resources or supports regarding mental health, mental health literacy, and ways to discuss acculturation and mental health during buddy sessions. Encourage mentors to help mentees leverage their cultural strengths through activities like sharing cultural knowledge, wellbeing practices, and identification of existing capacities
Removing systemic barriers to service access	Selective	Government, insurers, local health services	Formal support	Increasing service engagement by removing barriers such as high costs, extended wait times, or limited referral pathways	Policy changes and funding increases	Advocacy from representative groups such as student societies and mental health advocates. Other low cost, accessible tools such as culturally adapted telehealth or digital tools.
Increasing cultural appropriateness of mental health services	Selective	Education-based health services and other mental health service providers	Formal support	Increasing service engagement by adapting services to meet cultural needs of students	Cultural humility training for service providers, employment of culturally diverse workforces, and improving environments to help international students feel welcome and safe	Evaluations of current services and environments to identify possible local culturally relevant enhancements. Develop or draw upon assessment tools and therapeutic approaches that identify and build upon international students’ cultural strengths, existing capacities, and resilience factors
Academic and language supports	Selective	International students with poor grades or language issues	Improved academic performance	Reducing distress due to poor academic performance and associated individual or family expectations	Academic support services, “Study Buddies,” ESL programs, writing centers, communication workshops, and policies and procedures to identify performance issues and link students with support	Integration of culturally relevant mental health materials and support to help people manage distress related to learning difficulties. Identification of existing skills, capacities, or culturally based learning styles that could support improved academic performance
Peer work programs	Selective	International students accessing services	Formal support, social connection, and sense of belonging	Increasing service engagement through culturally relevant services and shared experiences. May also address loneliness and sense of belonging through group programs.	Trained peer workers, often integrated into education provider health services	Structured formalized training with ongoing feedback mechanisms for cultural adaptation and refinement. Integrate cultural strength approaches in training, delivery, and supervision of peer work
Culturally adapted risk screening tools	Selective	International students accessing services	Formal support	Increasing service engagement specifically for suicide through identifying those at risk who may not otherwise receive suicide-specific support	Risk screening tools that are culturally appropriate and accessible (e.g., student’s native language) that can be used to identify potential suicide risk	Drawing upon validated tools from other countries and cultures. Development of specific tools based on common risk factors for international students
Financial supports	Selective	International students experiencing financial difficulty	Financial security	Reducing distress associated with financial difficulty	Local programs or supports that allow international students to seek financial support	Communication of available supports via culturally relevant communication channels
Coping interventions	Selective	International students experiencing poor mental health or suicidality	Coping skills	Reducing suicide risk or suicidality through improving coping skills	Training programs or digital interventions targeting coping skills based on existing evidence-based approaches such as CBT and DBT	Culturally adapted CBT tools validated in non-western settings such as LMICs representative of student groups in host country. Integrate traditional coping strategies and cultural strengths into interventions. Develop approaches that leverage students’ existing cultural resources and resilience factors.
Specialized mental health providers	Indicated	International students with high risk levels for suicide through ideation with a plan or previous suicide related behavior.	Formal support	Increasing service engagement, retention, and outcomes through specialized providers for those who may not otherwise seek help	Employment of mental health providers specialized in working with international students, ideally with the same cultural background where possible	Working with specialized providers to help develop training and support structures for non-specialized providers
Crisis supports	Indicated	International students experiencing thoughts of suicide	Formal support	Increasing service engagement for those in crisis who may not otherwise seek help	Crisis support phone lines/chat services, emergency departments and other dedicated support available 24 h for international students	Training on common cultural risk and protective factors for suicide relevant to the most prevalent international student groups in the crisis support service country
Postvention strategies	Indicated	All staff, students, and community members affected by suicide	Help-seeking, formal support, effective crisis management, including safe communication	Reducing risk of suicide clusters and improving community safety in the wake of a suicide	Postvention plans, policies, and procedures with clearly outlined responsibilities and training for all implicated in the policies and procedures	Greater emphasis on cultural considerations in postvention planning and response.

CBT, Cognitive behavioral therapy; DBT, Dialectical behavioral therapy; LMIC, Low- and middle-income countries.

In summarizing current best practice recommendations in this way, several observations can be made. Firstly, most prevention recommendations focus on increasing formal support engagement either directly or through improving help-seeking, with a handful capturing other protective factors such as social connection, academic performance, and coping skills. The recommendations span across universal, selective, and indicated levels of prevention, with a notable emphasis on universal and selective approaches. Aligning with this, the mechanisms underpinning the majority of recommendations concentrate on engaging students — particularly those less likely to utilize services — by enhancing mental health literacy, customizing services for culturally diverse student populations, improving risk screening and referral (see AFSP Interactive Screening Program for a recent example), and overcoming structural barriers such as service costs and availability. Other mechanisms address specific risk factors such as loneliness, distress regarding academic or acculturation issues, and lack of effective coping skills. While most recommendations cater to international students, the roles of educational institutions, service providers, insurers, local communities, and families are also acknowledged. The delivery methods employed are varied, encompassing information dissemination and promotional activities, mental health initiatives such as stigma reduction campaigns, gatekeeper training to identify and respond to crises, peer support, and specialized support services addressing mental health and academic challenges. Importantly, in the final column, we highlight how each of these existing approaches could be improved through a culturally and contextually responsive approach.

Several core themes emerge amid the identified opportunities for improving existing prevention efforts using a culturally responsive approach. These encompass aspects such as community involvement and empowerment, a critical examination of the proposed mechanism of change and its foundational assumptions (e.g., western mental health views), and considerations for implementation and evaluation vital to developing or refining prevention programs. While we do not see these as an exhaustive list, they provide a valuable starting point for improving equitable suicide prevention efforts with international students. A set of questions we used to consider each of these areas is included in the Supplementary materials, and a more detailed explanation is provided below.

Community empowerment and, in some cases, ownership are widely acknowledged as central to improving mental health and suicide prevention efforts with migrants, minoritized groups, and youth populations (Gustavson et al., 2021; Reifels et al., 2021; Allen et al., 2022). In the case of existing programs and recommendations, an examination of current community involvement practices highlights numerous opportunities for enhancement. For example, moving from solely community participation to community empowerment could be used to adapt and improve gatekeeper programs by aligning them with students’ cultural context and real-world experiences. This could increase program acceptability, relevance, engagement, and efficacy, thereby raising the probability that the skills taught will be utilized (Nasir et al., 2016). Moreover, when communities possess some level of ownership and shared leadership over an initiative, it can enhance uptake and program sustainability and positively impact all community members involved (Blignault et al., 2023). Few existing prevention programs or recommendations include community empowerment at this stage, and this should be a focus for future efforts.

Mechanisms of change are also a central consideration in creating culturally responsive approaches. By examining the prevention strategy’s proposed mechanism of change, a multitude of refinement opportunities can also be identified to optimize effectiveness and cultural sensitivity. Building on the existing evidence with minoritized populations’, considerations could include whether the model acknowledges non-Western mental health perspectives, leverages students’ cultural strengths, and is flexible enough to integrate diverse cultural viewpoints and acculturation levels (Na et al., 2016; Bowden et al., 2020; Allen et al., 2022; Meza and Bath, 2022). For example, mental health literacy campaigns could draw upon non-Western views that underpin stigma, such as language (e.g., thinking too much) and use this to better engage students based on their existing perspectives before seeking to change their views (Kaiser et al., 2015). Alternatively, mental health services could adapt to such perspectives as part of making service provision culturally responsive, and promotion activities could then acknowledge how such perspectives are respected.

Implementation and evaluation are also key considerations in effective suicide prevention research and practice (Gustavson et al., 2021; Reifels et al., 2022). While little research has studied implementation in international student suicide prevention, insights can be gleaned from the broader field. A recent scoping review of 64 suicide prevention studies identified key implementation considerations: aligning efforts with community needs, interests, and settings; ensuring those responsible have relevant skills, knowledge, and resources; and engaging community leaders and organizations where possible when working with diverse groups (Kasal et al., 2023). A key consideration, in line with these findings, is who is best placed and should be responsible for delivering prevention efforts for international students. This involves determining who has the power to implement the prevention approach and the willingness to do so. As an example, the removal of systemic barriers to service access could be addressed by governments, insurers, or local health services, and an identification of which entity or combination thereof would be best placed and willing to do so in the local context is an important consideration when defining prevention approaches, policy, and systems (Jamieson, 2021).

Effective implementation paves the way for rigorous evaluation, the linchpin in assessing and refining our approaches. Effective evaluation demands rigorous empirical research employing validated measures or robust qualitative methods, incorporating value considerations, and interpreting findings within the context of current theories and literature (Coryn et al., 2017). Importantly, both prevention effort outcomes and implementation should be assessed (Reifels et al., 2022). This process facilitates vital feedback mechanisms to identify opportunities to enhance programs or services and their associated outcomes. For instance, these processes could help service providers understand why international students are likely to not engage in or drop out of services, providing a basis for subsequent improvements. Moreover, evaluation efforts should be culturally responsive, ensuring that the metrics and methods used are appropriate and meaningful for the diverse international student population being served (McKay et al., 2023).

Each of these overarching considerations, while separate, also have significant overlap and can inform the others. For instance, effective evaluation inherently depends on a degree of community engagement, and careful deliberation of the proposed mechanism of change is essential in determining the most suitable entity to implement programs and deliver the desired outcomes. Aligning with a Public Health Model of Suicide Prevention (Pirkis et al., 2023), adopting a framework that assesses approach, prevention levels, protective factors, mechanisms, target groups, and methods against cultural responsiveness perspectives can reveal significant opportunities to enhance current prevention strategies. This framework is also relevant to the identification of novel prevention strategies for international students.

### 2.2 Novel suicide prevention opportunities based on a culturally responsive approach

A strengths-based, culturally contextualized approach facilitates opportunities for novel prevention initiatives by highlighting alternate knowledge, perspectives, and needs along with associated strengths and protective factors that are not part of current approaches. We do not aim to present an exhaustive list of potential novel interventions here but instead highlight the ways the framework could facilitate the development of such approaches. As part of this, we draw upon existing evidence with other minoritized and migrant groups to demonstrate how such approaches may work.

There are numerous opportunities for novel prevention strategies for international students based on increasing community empowerment. Co-design and lived experience involvement are obvious starting points. There is a growing body of evidence highlighting the value of co-designed suicide prevention initiatives with young people and diverse communities (Thorn et al., 2020; O’Brien et al., 2021; Fitzpatrick et al., 2023). Lived experience involvement in developing new programs may support the identification of strengths-based prevention approaches better adapted to international students’ cultural expectations and needs. A review of mental health programs co-designed with culturally and linguistically diverse groups revealed that this collaborative approach was instrumental in creating programs more attuned to community needs (O’Brien et al., 2021). These programs commonly embraced peer support and group or family-based therapies to tackle challenges linked to individualistic and non-culturally adapted care models, fostering enhanced engagement and improved outcomes.

Similarly, co-design can support the identification of potential intervention strategies relevant to non-western explanatory models of mental health and community healing. For instance, evidence from several Culturally and Linguistically Diverse (CALD) communities in Australia shows that informal help-seeking through social, religious, and self-help is often preferred to formal help-seeking (Krstanoska-Blazeska et al., 2023). This is particularly true among ethnoracially minoritized college students in the U.S. (Lipson et al., 2018). Considering this, identifying typical informal mental health support sources used by international students and collaborating with them to co-design mental health support training tailored for their community could be highly beneficial. This could increase the capacity of relevant informal support sources to help international students facing mental health difficulties. Similarly, efforts could be made to improve links between these informal help sources and culturally adapted formal support services. Such a strategy could offer international students less daunting and more accessible care pathways than required when navigating formal support services alone. Initiatives to also increase college students’ agency in seeking mental health support may also increase their perceived cultural capital and improve their linkage to care (see Richards, 2022).

Another opportunity lies within international student communities and their shared experiences. While international students represent a diverse range of cultures, the shared identity, knowledge, and experiences of being an international student may provide a starting point for relevant prevention initiatives that traverse cultural divides (Gu et al., 2010). Designing suicide prevention initiatives around international students’ shared experiences can potentially yield programs relevant to a wider audience, thus diminishing the necessity for specific programs for each distinct cultural group of international students. For instance, international students often face challenges and stress related to acculturation when they move to a new country (Orygen, 2020). These acculturation experiences and the related acculturative stress are the central differentiating factor in the experience of international students and other student groups. Evidence suggests that acculturative stress increases suicidality in young people (Polanco-Roman et al., 2023). Psychoeducation programs have been found to be most effective in reducing acculturative stress in international students (Aljaberi et al., 2021), suggesting they could provide a strong foundation for future suicide prevention efforts centered on acculturation. Similar approaches addressing other shared experiences may also present other opportunities for novel prevention initiatives that are applicable to diverse international student groups.

Developmental considerations both generally and as part of the acculturation processes are also important for international student suicide prevention. For instance, cultural continuity and identity should be a primary focus, as students seek to negotiate who they are within a new cultural context, often free of their family and friend networks from their home country (Szabo and Ward, 2015; McKay et al., 2022). While these processes can foster youth development, they can also give rise to complex relationship dynamics when young individuals adopt perspectives differing from their parents’ and such acculturative tension has been identified as influencing suicidal behaviors (Gulbas and Zayas, 2015), this intergenerational acculturation conflict is particularly true among college students (Meza et al., 2024). Research and practice should seek to understand how such developmental processes impact suicidality and can be supported in such a way as to draw upon international students’ unique strengths to reduce suicide risk.

Finally, another key opportunity for improved suicide prevention with international students lies with digital interventions and tools. These tools can effectively integrate the essential components outlined in the ecological validity model, which offers specific considerations crucial for designing culturally responsive prevention and intervention approaches (Bernal et al., 1995). These include delivering the intervention in the participants’ preferred language, involvement of culturally relevant individuals in design and delivery, integration meaningful cultural metaphors, and a focus on ensuring the content reflects the participants’ cultural, social, and historical contexts. Furthermore, it stresses the alignment of psychological concepts, intervention goals, and methods with the participants’ worldviews, values, and needs, all within a comfortable and appropriate setting. Digital tools are well suited to providing adapted content through changing the language, metaphors, and imagery for different groups, all in a setting that may be more comfortable for international students than traditional in-person therapeutic models. For example, core mental health information or therapeutic tools could be delivered in students’ native languages, a preference commonly expressed by international students when engaging with digital mental health content (Choi et al., 2023).

Like the existing interventions, each of these opportunities may overlap. A new approach could involve co-designing a digital tool that incorporates shared acculturation experiences and developmental considerations with the intervention available in multiple languages, each incorporating culturally relevant metaphors and examples. Similarly, a complimentary approach could be taken where components of existing approaches (top-down approach) are combined with the new approaches to create novel (bottom-up approach) international student suicide prevention programs and interventions.

## 3 Discussion

Our exploration of current approaches and recommendations for international student suicide prevention, using our proposed extended Protective Factors Framework, has highlighted several critical themes for improvement: the importance of community empowerment and ownership in developing and implementing prevention strategies; the need to critically examine and adapt mechanisms of change to align with diverse cultural perspectives; and the significance of culturally contextualized implementation and evaluation processes. These themes build upon existing approaches in culturally contextualized suicide prevention while specifically addressing the unique context of international students in higher education settings (Allen et al., 2022; McKay et al., 2023).

Our findings extend the understanding of culturally contextualized suicide prevention by integrating the Protective Factors Framework with universal, selective, and indicated public health prevention levels, along with opportunities for strength-focused cultural adaptations. This approach offers a more nuanced understanding of how cultural factors interact with traditional risk and protective factors in suicide prevention for international students. It proposes a fundamental shift in conceptualizing and approaching suicide prevention, emphasizing cultural strengths and resources over deficit-focused models, aligning with recent trends in cross-cultural psychology and global mental health that advocate for more culturally aligned interventions (Chu et al., 2019; Bowden et al., 2020).

While this framework provides a broad structure for culturally responsive suicide prevention, we also must acknowledge the inherent complexity of addressing the needs of such a diverse population. International students come from a wide array of countries and cultures, each with its own unique perspectives on mental health, help-seeking behaviors, and suicide (Na et al., 2016; Veresova et al., 2024). This diversity presents both challenges and opportunities. The heterogeneity within the international student population poses significant challenges in developing universally applicable strategies. What works for students from one cultural background may not be effective or culturally appropriate for others. This diversity necessitates a careful balance between creating a broad, inclusive framework and ensuring that interventions can be tailored to specific cultural subgroups. Additionally, the lack of existing evidence-based programs specifically for international students limits our ability to fully assess the effectiveness of the proposed enhancements (McKay et al., 2023).

However, this diversity also presents unique opportunities. The framework’s emphasis on community empowerment and cultural strengths allows for flexibility in implementation. It encourages the development of interventions that can be adapted to various contexts and groups while maintaining core principles of culturally contextualized prevention. Moreover, the shared experience of being an international student, despite differing cultural backgrounds, provides a common ground for developing interventions that address universal challenges such as acculturation stress, language barriers, adapting and learning how to navigate new social systems (i.e., health and education systems) and academic pressures in a new environment.

It is crucial to also acknowledge that international students’ experiences can vary significantly based on their host country and the specific educational system in which they are studying. Major destination countries like the U.S., Australia, and the UK have distinct cultural, social, and educational environments that can profoundly impact students’ experiences, including their acculturation stress and mental health outcomes (Smith and Khawaja, 2011). Additionally, international students may face challenges related to racism, sexism, and other forms of discrimination in their host countries, which can significantly affect their mental health and increase suicide risk (Wang et al., 2013; Taliaferro et al., 2020; Pérez-Rojas et al., 2021). The intersectionality of being an international student with other social identities (e.g., race, gender, sexual orientation) and its interaction with structural systems (e.g., education and health) can further complicate these experiences (Herridge et al., 2023). This may be compounded by students experiencing minority status for the first time, necessitating a re-evaluation of their racial/ethnic identity within unfamiliar social hierarchies (Fries-Britt et al., 2014). These complex processes of identity development and navigation of new cultural and educational contexts necessitate more nuanced prevention approaches that account for these diverse influences and their potential interaction with suicide risk.

Future research should focus on empirically testing culturally contextualized adaptations across different international student subgroups and host countries, conducting longitudinal studies to assess long-term impacts, exploring intersectional impacts, and investigating the potential of tailored digital mental health interventions. Studies on effectively implementing and scaling culturally responsive interventions in diverse university settings are also needed. Additionally, research should explore how this framework can be effectively adapted for different cultural subgroups within the international student population, identifying both universal elements that apply across cultures and specific components that need cultural tailoring.

Despite these limitations, the proposed model presented here contributes significantly to the current understanding of culturally responsive suicide prevention. By providing a structured framework for assessing and enhancing existing approaches, it offers a comprehensive tool for researchers and practitioners working with international students or other diverse groups. This approach not only highlights ways to improve current strategies but also identifies opportunities for novel culturally grounded interventions that leverage the unique strengths and experiences of those people they are seeking to help. It is our hope that this work will inspire continued research and development in this field, leading to more effective and culturally attuned suicide prevention interventions for international students and other culturally diverse at-risk populations.

## Author contributions

SM: Conceptualization, Formal analysis, Methodology, Writing – original draft, Writing – review & editing. JM: Conceptualization, Formal analysis, Methodology, Writing – review & editing.
