# Supplementary material for: Culturally contextualized suicide prevention for international students: new opportunities for research and practice
Source: Front Psychol. 2024 Aug 6;15:1418185. doi: 10.3389/fpsyg.2024.1418185 (PMC11333360; doi:10.3389/fpsyg.2024.1418185)
Supplement: Supplementary file 1 [file Table_1.DOCX]

Questions to Enhance Cultural Contextualized Suicide Prevention Strategies for International Students

## Community involvement

1. Whose voices should be given priority in developing prevention efforts and why?
2. Can international students take ownership in the development and implementation of the prevention approach? If not, are there ways to increase international student involvement/ownership?
3. What strategies can be employed to ensure that community consultation is meaningful and informs prevention efforts? Who should be included in this process (e.g., student groups, community organizations, education providers, service providers etc.)?

## Mechanisms/Model of change

1. Does the approach operate at a universal, selective, or indicated level?
2. Does the approach seek to meet the cultural needs of students, or does it require cultural adaptation on the part of the students?
3. What methods will be employed to identify and integrate students' cultural strengths into the suicide prevention approach?
4. Does the approach accommodate varying levels of acculturation among international students and the ways this may impact views of suicide, help-seeking, and treatment?
5. Can the model of change be adapted to include culturally specific notions of suicide without imposing a one-size-fits-all standard?
6. Is there evidence from other similar communities that could guide development or refinement?

## Implementation

1. Would a local health system, education provider-led, community-based, or another type of delivery model be the best option to meet international student needs?
2. Who has the power to implement the program, policy, or funding changes, and are they willing to make such changes?

## Evaluation

1. What feedback mechanism is necessary to ensure the prevention approach is and continues to be culturally relevant?
2. How can evaluation materials, including consent documents, be made accessible and acceptable to participants?
3. Is there an opportunity for the evaluation process to be a tool for empowering international students and validating their experiences?
